# Investigation of the effectiveness of a new portable ‘cryopen’ probe for cryotherapy (CryoTreq®)

**DOI:** 10.1186/s40942-021-00302-y

**Published:** 2021-04-07

**Authors:** Peter Stalmans, Joris Vander Mijnsbrugge, Filip D’Hollander, Rita Van Ginderdeuren

**Affiliations:** 1 Dept. Ophthalmology UZ Leuven, Herestraat 49, 3000 Leuven, Belgium; 2 Onze-Lieve-Vrouwziekenhuis, Aalst, Moorselbaan 164, 9300 Aalst, Belgium

**Keywords:** Cryopexy, Cryotherapy, Cryotreq, New vitrectomy products, Retinal detachment, Retinal choroidal scars

## Abstract

**Background:**

To test the effectiveness of a new, innovative, single-use handheld ‘cryopen’ (CryoTreq®), which has been developed and marketed by Vitreq.

**Methods:**

Animal testing of cryopexy using the Cryotreq ® in two pig eyes was performed.

**Results:**

The biological effects of cryopexy were observed.

**Conclusions:**

During the surgery, the handheld device was comfortable to use and offers the surgeon more freedom to move. The cryotherapy caused severe atrophy and thinning of retinal layers, pigment epithelium and choroidea in a relatively uniform thickness. The cryopen is effective and offers advantages over non-handheld cryotherapy devices.

## Background

Retinal cryopexy (also known as retinal cryotherapy) is a procedure that utilizes intense cold to stimulate the formation of a chorioretinal scar. Cryopexy destroys retinal and choroidal tissue. This freezing treatment is an important technique used either as a procedure on its own or as part of vitrectomy surgery. Specifically, it is used to fuse and seal the retina against the eye wall. The eye surgeon uses a specially designed freezing probe to apply intense cold (down to around − 80 °C − 90 °C) to freeze the area of the retina around a retinal tear or detachment. This procedure is performed under local or general anesthesia.

Early attempts at cryopexy were first seen in the early twentieth century. This was when Deutschmann used solid carbon dioxide snow to achieve ultra-low temperatures [1]. Bietti used a mixture of this and acetone to reach a temperature of -80 °C and induce adhesive choroiditis [2].

Thirty years later, cryopexy was used in the removal of cataracts [3]. In this procedure, alcohol and solid carbon dioxide were used as a cooling mixture. And in 1963, chorioretinal scars were induced in rabbits using a cryosurgical unit that was designed to treat neurological movement disorders and utilized liquid Nitrogen [4]. This could achieve ultra-low temperatures of around − 196 °C.

In 1964, Lincoff et al. introduced their own design for a probe for trans-scleral treatment of retinal diseases that produced temperatures of − 90 °C. In this instance, the probe was designed to operate from a Cooper-Linde cryosurgical unit.

In this time period, experimental work in animals and early experience in humans indicated that temperatures of − 20 °C to − 40 °C were required as a maximum for effective clinical use. Humans were first treated with cryopexy in an ophthalmology setting in 1963 by Lincoff, who reported the following year on his first 30 cases of treatment of retinal tears with or without retinal detachment by cryopexy [5]. Lincoff observed that cryotherapy did not cause scleral complications, such as those seen following diathermy application to full- thickness sclera. This supported a transition from diathermy to cryotherapy for retinal detachment repair in general clinical practice. Subsequently, smaller, lighter, less- complicated instruments for cryopexy that are safe and can be easily maintained were developed that employ the Joule–Thomson effect in cooling gases, such as Nitrous Oxide (N_2_O) or Carbon Dioxide (CO_2_) [6].

Cryopexy today involves use of a specially designed, metal cryotherapy probe. Probes are often operated through a foot pedal. When the foot pedal is pressed, the tip of the cryopexy probe cools due to the rapid expansion of very cold gases (usually N_2_O) within the probe tip. When the probe is placed on the eye, tissue destruction is induced by the formation of water crystals followed by rapid thawing results. Healing and the formation of scar tissue ensues.

In the case of retinal detachment, the tissue around each of the retinal tears is targeted. Cryopexy stimulates scar formation, sealing the edges of the tear. The surgeon usually oversees the procedure through an indirect ophthalmoscope, while pressing gently on the outside of the eye using the cryopexy probe, producing a small area of freezing that involves the retina and the tissues immediately underneath it. Using multiple small freezes like this, each of the tears is surrounded. The agitated tissue forms a scar, which brings the retina back into contact with the tissue underneath it. More than one cryopexy procedure is often required to repair retinal damage.

It is important to note that in humans it is quite common to perform bilateral cryotherapy in one session. Cryotherapy in humans is usually done under retrobulbar or less commonly (in children) under general anesthesia, afterwards no prolonged pain medication is required. An injection with steroids is given around the eye at the end of the surgery to reduce inflammation. There is only contact between the outside of the globe and the cryoprobe, no incision is made in the eye. Hence, there is no risk of intra-ocular infection. There are some risks of complication involved in cryopexy, although they are quite uncommon. They include perforation of the eye with the anesthetic needle, bleeding, double vision and glaucoma.

In practical terms, cryotherapy devices are currently often about the size of a shoe box, and cool through the expansion of N_2_O or CO_2_ gas. Therefore, a gas bottle is often attached to the device. The cryoprobe itself is connected to the device via a cable of about 2 m in length. Within this cable is a small capillary semi-rigid tube, through which the gas flows to the tip of the probe and is expanded there. This type if construction has as disadvantage that the unit is relatively less mobile, and that the capillary tube in the cable easily nicks or gets clogged. As a result, these types of devices can fail and require repairing.

The device used in this study that circumnavigates these issues. The cryopen is preloaded with N_2_O gas. In contrast to many other commercially available cryopexy devices, this new device is hand-held and hand-operated rather than foot-operated. It achieves a rapid freeze in three seconds, has a fixed tip, and is ready-to-use, with no set-up or priming required.

The purpose of this study was to test the effectiveness of the handheld cryopen in an in vivo animal model.

## Materials

The ‘cryopen’ (Cryotreq ®) used in this study is manufactured by Vitreq (Vierpolders, The Netherlands) and is a stand-alone, single-use, disposable cryopexy device. It is approximately 20 cm long and a few cm thick without a connection to another device. Internally, for the required gas expansion, N_2_O patterns are used similar to those used in espuma-devices found often in professional catering.

Minimally 15–20 cryopexy spots can be delivered in the lifetime of one disposable device. Its tip is cooled to approximately − 88.5 °C (when not in contact with tissue). The internal cartridge consists of 7.5 g of N_2_0 in a volume of 10 ml. The cryopen is sterilized using gamma radiation and packaged in a Tyvek pouch. It also includes a gas exhaust tube, which safely transports the gas used in the device to below operating height.

For further details on the design and specification of Vitreq’s ‘CryoTreq®’, see Appendix 1.

### Animal testing

An experiment on a single, live porcine animal was designed to show the effectiveness of the cryopen as the biological effects of cryopexy are quite obvious and distinct (fibrosis and atrophy of the retina and underlying areas). Since these effects are only visible after 10–14 days, the biological effects of cryopexy cannot be examined on *post-mortem* eyes. Hence, it was necessary to employ an animal in animal testing that remained alive after the treatment. Pigs’ eyes are approximately the same size as a small human eye. For these reasons, pigs were selected as the species most appropriate for animal testing in this instance. One domestic pig (Sus scrofa domesticus) of a weight of 50 kg was treated in both eyes with cryotherapy using Vitreq’s handheld device.

The ethical considerations and ethical protocol developed in part by the UZ Leuven Ophthalmology Research Group and aligned with EU animal welfare standards were approved by the Medanex Ethical Committee and applied rigorously during the test.

## Methods

The biological effects of cryotherapy (fibrosis and atrophy) were examined. A successful cryopexy treatment always induces fibrosis and atrophy at the retina.

The pig was treated according to the following protocol:

- General anesthetic
- Photographic documentation of the retina
- Application of cryo-coagulates in both eyes under ophthalmologic guidance.
  Duration of the coagulates
  - 1 second
  - 2 seconds
  - 4 seconds
  - 8 seconds
  - 16 seconds
  - 32 seconds
  - 64 seconds
  The last times are quite long, in a human eye the application time is only a few seconds. However, the porcine sclera is about three times thicker than in humans, hence a longer required application time was expected to create tissue scarring.
- Cryopexy treatment
  Use of the Vitreq ‘cryopen’ in cryopexy procedure was according to the manufacturers’ guidelines, as below:
  1. No set-up or priming time for the device was required as it was ready-to-use.
  2. The device was held, and the tip moved to the desired cryopexy location.
  3. The button on the device was pressed to activate the cryopexy functionality.
  4. The button on the device was released to stop activation of the cryopexy functionality.
  5. Steps 2–4 were repeated as necessary.
- Parabulbar injection of triamcinolone
- Eye ointment: Maxitrol (Alcon, Forth Woth, TX, USA), containing Dexamethasone, Neomycinesulphate and Polymyxine B-sulphate
- Two weeks free running around, no need for postop medication
  After this, 14 days postoperative follow-up was performed, and a final evaluation was made through in vivo and post-mortem eye examination as follows:
- General anesthesia
- Photographic documentation of the retina
- Enucleation of the eyes, fixation for pathological examination
  Following enucleation, the pig’s eye was fixated in 4% Formalin for one week. The eye was surgically opened according the smallest axis. After visualization of macroscopic scars, two sections were made through the largest diameter of the scar. The section was routinely processed to paraffin, 5 mm section were cut and after dissolution of the paraffin, stained with Hematoxylin and Eosin.
  Microscopy of the sections was performed with a Leica DFC290 HD imaging camera (Leica, Wetzlar, Germany)
- Euthanasia

## Results

During the surgical treatment, the CryoTreq® was comfortable to use. The compactness of the pen format and absence of a semi-rigid connecting cable to a head device made the cryopexy treatment easier and more accurate since the cryopen offers the surgeon more motion freedom. Even though very long cryocoagulation was performed, one device was sufficient per eye treated.

During the two weeks after the treatment, no abnormal behavior or indication of pain was observed.

**Fig. 1:**
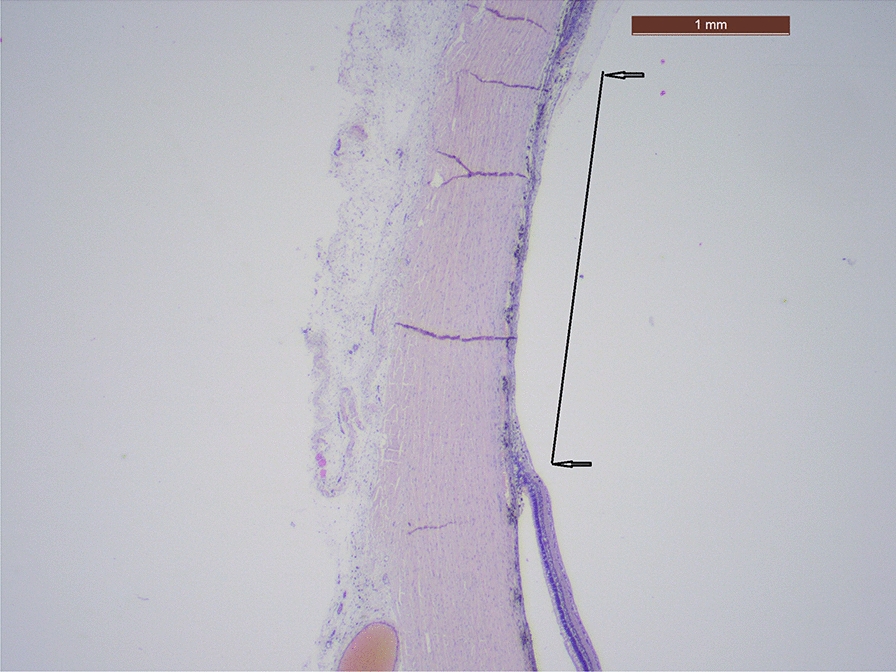
Pathological section of the retina after treatment with the CryoTreq® probe. Low magnification view of largest retinal and choroidal scar (between 2 black arrows) of the left eye of the pig. Deep atrophic layers are visible. Hematoxylin & Eosin stain, bar = 1 mm

**Fig. 2:**
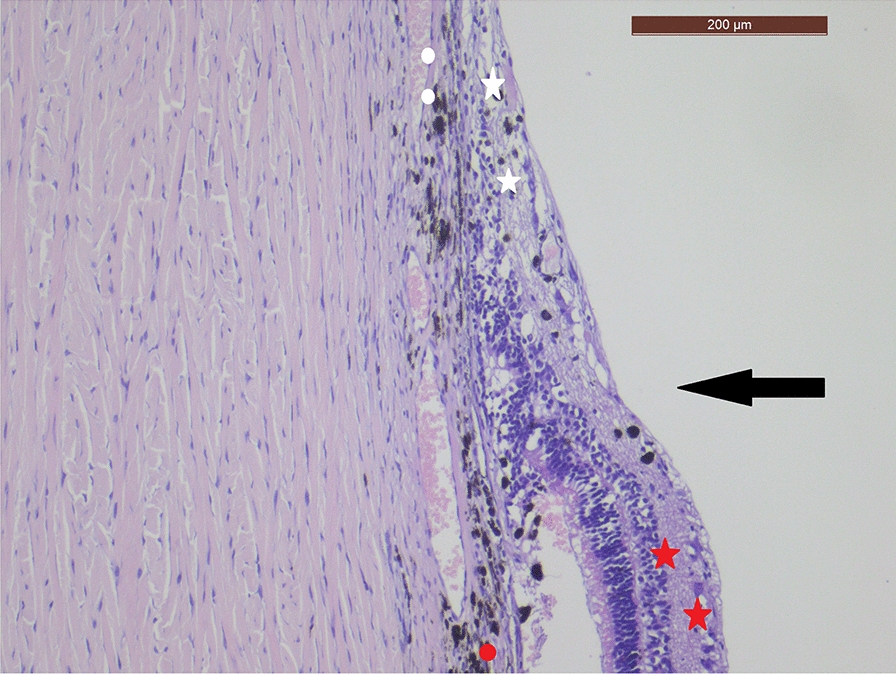
Pathological section of the retina after treatment with the CryoTreq® probe (magnified). Higher magnification of abrupt transition from normal retina (bottom of picture, stars) to scar tissue (circles), indicated by large black arrow. All retinal layers are atrophic; retinal pigment epithelium has disappeared; only large vessels and clusters of pigmented melanocytes are left in the choroid (red circle). Hematoxylin & Eosin stain, bar = 200 μ

Pathological examination showed that the pig’s normal retinal layers (250 microns) abruptly ended in the zone treated with the cryopen (Figs. 1 & 2). The photoreceptor layer had disappeared. All retinal layers showed atrophy with a 20 micron thickness as the core end-result. The retinal pigment layer was absent. In the choroidea, the choriocapillaris layer was extremely thinned; only large blood vessels and clumps of pigmented melanocytes were present after cryopexy treatment.

The eye was fixated in Formalin 4% for 1 week. The eye was opened following the smallest axis. After visualization of macroscopic scars, 2 sections were made through the largest diameter of the scar. The section was routinely processed to paraffin, 5mm section were cut and after dissolution of the paraffin, stained with Hematoxylin and Eosin. Microscopy of the sections was performed with an imaging Leica DFC290 HD camera. The normal retinal layers (250microns) abruptly ended in the treated zone; the photoreceptor layer disappeared, all retinal layers showed atrophy with 20micron thickness as central end result. The retinal pigment layer was absent. In the choroidea the choriocapillaris layer was extremely thinned; only large blood vessels and clumps of pigmented melanocytes were present after treatment. Conclusion of microscopy of the lesion after treatment: severe atrophy and thinning of retinal layers, pigment epithelium and choroidea

## Discussion

Cryotherapy tools for ophthalmology have remained on a plateau in terms of limited innovation for some time.

As most cryotherapy devices are not handheld, the tested cryopen provides a useful new and effective innovation for the ophthalmology surgeon’s range of options in cryopexy.

**Fig. 3:**
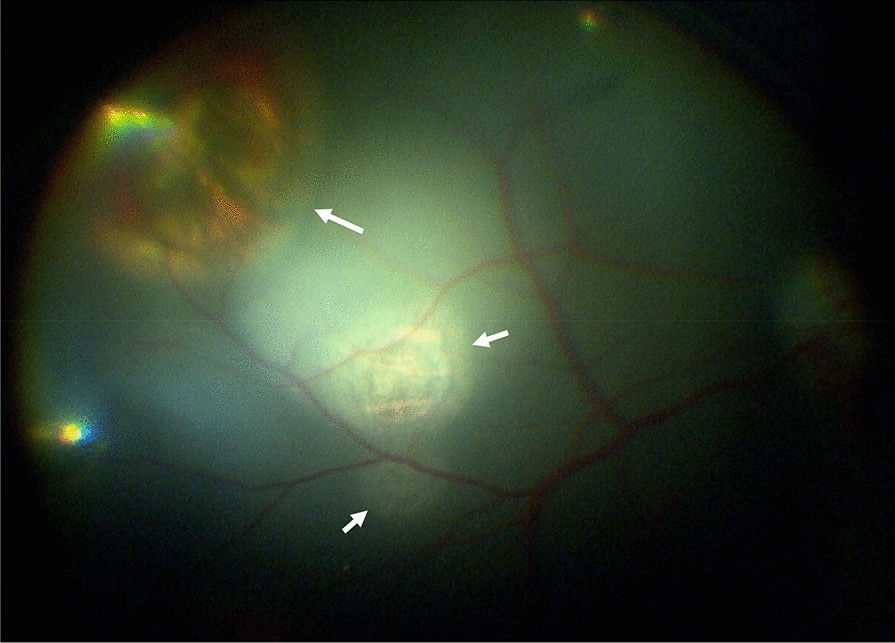
Retinal photograph taken two weeks after the cryotherapy with the CryoTreq® probe. Fundoscopy image showing the typical retinal atrophy with surrounding hyperpigmentation after cryotherapy treatment with the CryoTreq® Device

The treatment was performed under general anesthesia. Cryo-coagulations were placed under ophthalmoscopic guidance in the retinal periphery, hence they did not affect the central vision (Fig. 3). A (rare) complication of cryotherapy is local necrosis of the retina, which can induce a secondary retinal detachment, usually months to even years after the treatment. In testing, since euthanasia was performed after two weeks, there was virtually no risk of this complication.

## Conclusion

In summary, the treatment with the CryoTreq®, caused extensive atrophy and thinning of retinal layers, pigment epithelium and choroidea in a relatively uniform thickness. These lesions are the expected outcome after application of cryotherapy. The cartridge in the device was more than adequate to supply the freezing charges necessary to perform retinal cryopexy. Considering that the device can provide at least 15 freezing events, this is sufficient to perform a wide range of ophthalmic cryotherapies besides cryopexy, including, but not limited to, cyclo destructive procedures in refractory glaucoma, trichiasis and retinopathy of prematurity (ROP).

## Figures and Tables

**Fig. 4:**
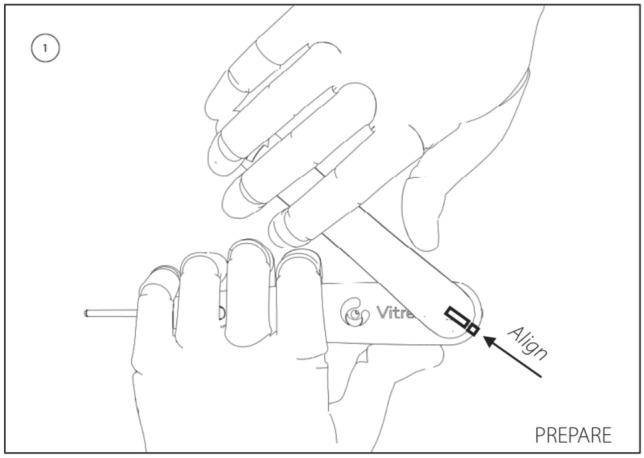
Preparation for activation

**Fig. 5:**
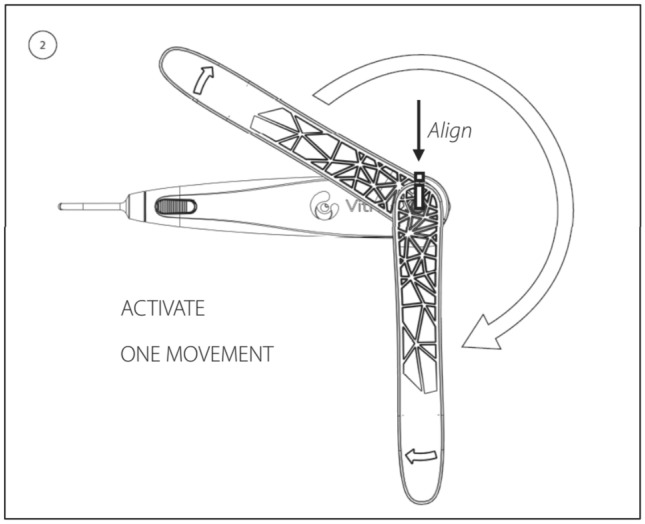
Activation

**Fig. 6:**
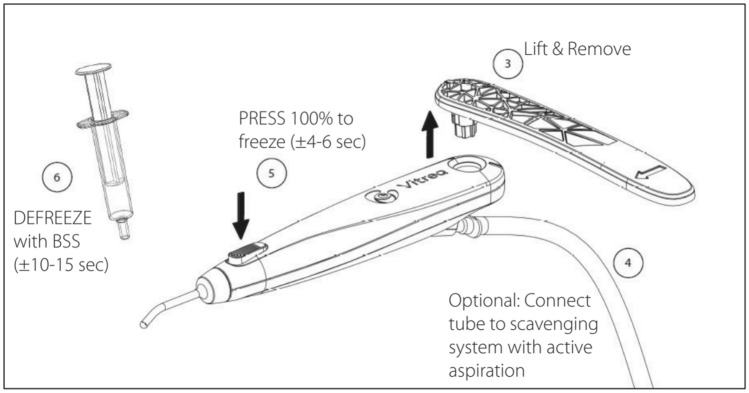
Finalize preparation & use

## Data Availability

Included in the article.

## Appendix 1: Specifications of the CryoTreq ® Device (Vitreq)

### Dimensions

- Length:176 mm
- Width:28 mm
- Height:37 mm

- Tip diameter:3 mm
- Tip length:30 mm
- Tip angle:32°
- Tip radius:10 mm

### Specifications

- The Cryotreq is a standalone cryo device.
- The Cryotreq is a single-use disposable device.
- The Cryotreq can place 15–20 cryo spots.
- Its tip is cooled to approximately − 88.5 degrees Celsius (when not in contact with tissue).
- The internal cartridge consists of 7.5 g of N20 in a volume of 10 ml.
- The Cryotreq is sterilized using gamma radiation and packaged in a Tyvek pouch.
- The Cryotreq includes a gas exhaust tube which can be connected to a scavenging system to transport the gas from the use environment.

### Usage Steps

#### Preparation

1. Please visually inspect the packaging of the sterile CryoTreQ for possible damages and expiration date. Devices from previously opened or damaged packaging or devices past the expiration date must be deemed unsterile and discarded.
2. Unpack the CryoTreQ for introduction into a sterile environment.
3. The CryoTreQ is intended to be used in a well-ventilated room with a downflow system (at least an air refreshment rate of 20 times per hour). If this is not available, attach the hose to the exhaust of the CryoTreQ and a scavenging system.
4. Have a syringe with sterile Balanced Salt Solution (BSS) at room temperature (22 °C or 72 °F) ready. BSS may be used to speed up the release of the tip after freezing.
5. Rotate the lever in a fluent motion in the direction indicated on the lever to prepare the CryoTreQ. Note: This may require a limited amount of force. After rotating the lever over 270 degrees and aligning the stripes on the lever and the tool (see Fig. 2), the lever can be manually removed by pulling it upwards (Figs. 4, 5 and 6).

Always check proper functioning of the CryoTreQ without tissue contact to the tip of the probe. Shortly (less than 2 s) push the activation button of the CryoTreQ, while pointing the tip downwards. The point of the tip should become white from the drop in temperature.

### Directions for Use

1. The patient’s eye should face the ceiling, to operate the CryoTreQ in the correct vertical position.
2. Anesthesia is used in cryotherapy.
3. When necessary, create an incision through the conjunctiva.
4. Insert the tip of the CryoTreQ to the area to receive cryotherapy on the exterior surface of the eye.
5. Activate the CryoTreQ until coagulation is visually successful.
6. Release the activation button to stop the freezing cycle.
7. Do not exert a pulling force before the tip is defrosted. Defrost by flushing BSS.
8. The CryoTreQ can be retracted and repositioned to the next area to receive cryotherapy.

The liquid gas container has a volume to use the CryoTreQ for approximately 15 freezing cycles at the same patient. The CryoTreQ shall be operated between 15 and 25 °C (59 to 77 °F). Above 25 °C (77 °F) a reduced performance may be observed: device is still safe and will still function but may not be able to generate specified amount of freezing actions.
